# The Association of Polymorphisms in Genes Encoding Antioxidant Enzymes GPX1 (rs1050450), SOD2 (rs4880) and Transcriptional Factor Nrf2 (rs6721961) with the Risk and Development of Prostate Cancer

**DOI:** 10.3390/medicina58101414

**Published:** 2022-10-09

**Authors:** Milica Djokic, Tanja Radic, Veljko Santric, Dejan Dragicevic, Sonja Suvakov, Smiljana Mihailovic, Vesna Stankovic, Milica Cekerevac, Tatjana Simic, Marina Nikitovic, Vesna Coric

**Affiliations:** 1 Institute of Oncology and Radiology of Serbia, 11000 Belgrade, Serbia; 2 Institute of Mental Health, 11000 Belgrade, Serbia; 3 Clinic of Urology, University Clinical Center of Serbia, 11000 Belgrade, Serbia; 4 Faculty of Medicine, University of Belgrade, 11000 Belgrade, Serbia; 5 Division of Nephrology and Hypertension, Mayo Clinic, Rochester, MN 55902, USA; 6 The Obstetrics and Gynaecology Clinic Narodni Front, 11000 Belgrade, Serbia; 7 Department of Pathology, University Clinical Centre of Serbia, 11000 Belgrade, Serbia; 8 Institute of Medical and Clinical Biochemistry, Faculty of Medicine, University of Belgrade, 11000 Belgrade, Serbia; 9 Serbian Academy of Sciences and Arts, 11000 Belgrade, Serbia

**Keywords:** reactive oxygen species, oxidative stress, antioxidant enzymes, prostate cancer, SNP

## Abstract

*Background and Objectives:* Mounting evidence implicates oxidative damage in prostate carcinogenesis, contributing to modifications of macromolecules that drive cellular malignant transformation. Functional single-nucleotide polymorphisms (SNPs) of enzymes involved in redox homeostasis can disrupt pro-oxidant–antioxidant balance, leading to accumulation of reactive oxygen species and oxidative damage. We investigated the potential role of genetic polymorphisms of antioxidant enzymes glutathione peroxidase 1 (*GPX1* rs1050450) and superoxide dismutase 2 (*SOD2* rs4880) and regulatory antioxidant protein nuclear factor erythroid 2-related factor 2 (*Nrf2* rs6721961) in the susceptibility to prostate cancer development (PC) and prognosis. *Materials and Methods:* We conducted a case–control study consisting of 235 patients with PC and 240 controls. Gene polymorphisms were determined by quantitative polymerase chain reaction (qPCR) and polymerase chain reaction with confronting two-pair primers (PCR-CTTP) methods. Multiple risk models were composed to inspect the separate and mutual effect of multiple genes and in combination with acquired contributory factors on the risk of PC development. *Results:* Independently, carriers of at least one SOD2*C allele had increased risk of PC development, which was significantly further amplified in advanced statistical models. When tested in combination, individuals with both SOD2*C allele and Nrf2*C/C genotype were also at increased risk of PC development, which was augmented when combined with acquired contributory factors. During the mean 75 ± 25 months of follow-up, investigated gene polymorphisms did not affect overall survival. *Conclusion:* Our results suggest that these gene polymorphisms could be used as risk biomarkers of PC evolution.

## 1. Introduction

In developed Western countries, prostate cancer (PC) remains one of the leading cancer-related causes of morbidity and mortality [1]. Excluding age, ethnicity and family history of PC, risk factors contributing to its development are poorly understood. Furthermore, due to PC heterogeneity, differentiation between indolent and aggressive phenotypes poses a challenge, which leads to overdiagnosis, overtreatment and decreased quality of life. In order to help tailor personalized therapeutic strategies for individuals with higher risk of worse outcomes, a variety of studies in recent years have been focused on determining possible biomarkers for risk stratification regarding PC development and survival [2].

Reactive oxygen species (ROS), such as superoxide (O_2_^−^) anion, hydrogen peroxide (H_2_O_2_) and radical (OH^−^) anion are common byproducts of aerobic cellular metabolism. They are constantly formed and reduced to less reactive compounds by various enzymatic and non-enzymatic mechanisms. A mounting body of evidence supports the role of ROS in prostate cancer evolution [3,4,5]. It is believed that during chronic inflammation preceding tumorigenesis, the balance of chemokines and cytokines, as well as ROS, is altered [6,7,8]. The accumulation of intracellular ROS leads to modification of macromolecules, including DNA and proteins. Moreover, ROS seem to serve as signaling modifiers by inducing glutathionylation, S-nitrosylation and formation of disulfides of proteins, thus changing their regulatory roles [9]. The aforementioned mechanisms are thought to be implicated both in the onset and progression of complex diseases, such as cancer [10]. The expression of certain regulatory antioxidant proteins, as well as antioxidant enzymes, also seems to be altered, contributing to the transformational potential of the affected cell [11,12]. In addition, genetic polymorphisms found in genes encoding for both regulatory antioxidant proteins, such as Nrf2 (Nuclear factor erythroid 2-related factor 2), and antioxidant enzymes may aid in tumor development and progression [13].

Transcriptional factor Nrf2, a key controller of antioxidant response, is directly regulated by ROS levels [14,15]. Under basal conditions, the level of Nrf2 expression is low. However, in stressed cells, Nrf2 induces the expression of various target genes involved in various cellular processes, such as redox homeostasis, xenobiotic metabolism, DNA repair, energetic metabolism and proteasomal degradation, among others [16]. Recent studies have revealed the dual role of Nrf2 in cancer evolution. The protective role of Nrf2, consisting of quickly inducing target genes and repairing cellular oxidative damage that originates from carcinogens and radiation, has been well established [17,18,19]. It is also proposed that its antitumor effect is achieved by inhibiting NF-kB mediated proinflammatory pathways [20]. However, in already formed cancer cells, Nrf2 activation can further promote cancer proliferation and contribute to radio- and chemoresistance [21,22,23]. Few functional polymorphisms of the *Nrf2* gene have been described in the literature, and their impact on various cancers is yet to be fully identified. Among discovered polymorphisms, *Nrf2* rs6721961 is one of the more frequently described. It contains a substitution of C to A in the gene’s promoter region at position -617. It has been suggested that this change affects basal expression of the *Nrf2* gene and may diminish transcription of its target genes [24].

The cornerstones of the enzymatic defense system for scavenging and neutralizing ROS are the superoxide dismutase (SOD) and glutathione peroxidase (GPX) families of enzymes. O_2_^−^ is reduced to H_2_O_2_ in a reaction catalyzed by SOD, and among the three identified isoenzymes, SOD2 is the most widely studied. In vitro studies have shown that SOD2 overexpression results in a reduced growth rate of androgen-independent prostate cancer cells [25]. Furthermore, it has been reported that SOD2 acts as a mediator, regulating various activities of transcriptional factors [26,27]. Multiple single-nucleotide polymorphisms (SNPs) have been discovered within the *SOD2* gene; however, the most extensively researched is substitution of C → T altering alanine to valine at position 16 of the amino acid chain (*SOD2* rs4880). This causes a change in the secondary protein structure, which in turn affects import of SOD2 into mitochondria, resulting in reduced activity of the Val variant [28]. Previous population studies have shown an association between this polymorphism and prostate cancer development [29,30,31]. The catalytic role of another main antioxidant enzyme, GPX, is to balance intracellular levels of H_2_O_2_ by further reducing it to H_2_O. Eight isoenzymes have been identified, among the most researched of which is GPX1. Within the *GPX1* gene, the most commonly studied polymorphism involves substitution of C → T shifting proline to leucine at position 198 (*GPX1* rs4050450). This modification causes conformational change, affecting enzyme activity, which can lead to increased cancer risk [32,33,34].

The aim of our study was to investigate the possible role of functional polymorphisms of antioxidant enzymes SOD2 and GPX1 and regulatory antioxidant protein Nrf2, alone or combined, in susceptibility to prostate cancer development and overall survival in Serbian male patients.

## 2. Materials and Methods

### 2.1. Selection of Patients

This case–control study enrolled 235 patients with initially localized prostate cancer who were diagnosed and treated at the Urology Clinic of the Clinical Center of Serbia in Belgrade and the Institute of Oncology and Radiology of Serbia from 2013 to 2016. Criteria for enrollment were an age of 18 years or older, histological confirmation of prostate adenocarcinoma by an experienced uropathologist in accordance with the WHO classification [35], physical examination and transrectally guided biopsy and voluntary acceptance to participate in the study prior to the start of the treatment. Information about demographic characteristics, medical history, diagnostics and treatment of prostate cancer was collected from standard questionnaires filled in by patients and from their medical records. The follow-up period for patients with prostate cancer was up to 98 months (from January 2014 to March 2022). The control group consisted of 240 age-matched males with no previously recorded malignancy whose DNA samples are a part of the DNA biobank at the Institute of Medical and Clinical Biochemistry, Faculty of Medicine, University of Belgrade. By selecting control subjects from the same population as the cases, the confounding effect of geographical factors or ethnic background was limited. Informed consent was obtained from all participants. The study was conducted according to the Declaration of Helsinki, the study protocol was approved by the Ethics Committee of the Faculty of Medicine, University of Belgrade (no.1322/XII-15).

### 2.2. Histological Assessment

Prostate adenocarcinoma was histologically confirmed by an experienced uropathologist in accordance with the WHO classification [35]. The histological assessment comprised the determination of Gleason score by the same dedicated uropathologist. Images indicating the most representative examples of histological findings of Gleason grades, ranging from 1–5, are provided in the Supplementary Materials (Figures S1–S4).

### 2.3. Laboratory Analysis

Serum prostate-specific antigen (PSA, ng/mL) concentration in patients diagnosed with prostate cancer was analyzed by CMIA (Cobas, Roche Diagnostics, Rotkreuz, Switzerland) as a part of routine laboratory work in the laboratory of the University Clinical Center of Serbia.

Whole blood was used to isolate genomic DNA with a QIAamp DNA mini kit (Qiagen, #51306, Chatsworth, CA, USA) in accordance with the manufacturer’s instructions. *GPX1* (rs1050450) and *SOD2* (rs4880) gene polymorphisms were determined by quantitative polymerase chain reaction (qPCR) on a Mastercycler ep realplex (Eppendorf, Hamburg, Germany) using applicable Applied Biosystems TaqMan drug metabolism genotyping assays (Life Technologies, Applied Biosystems, Carlsbad, CA, USA). For the *SOD2* polymorphism, a C_8709053_10 assay was used, whereas in case of the *GPX1* polymorphism, a custom-designed assay was applied with sequences 5′ VIC-ACAGCTGGGCCCTT-MGB-3′ and 5′ FAM-ACAGCTGAGCCCTT-MGB-3′. The *Nrf2* (rs6721961) gene polymorphism was detected by polymerase chain reaction with confronting two-pair primers (PCR-CTTP) method, as previously described by Shimoyama et al. [36]. Amplification products were separated on 2% agarose gel by electrophoresis and visualized using a UV ChemiDoc camera (Bio-Rad, Hercules, CA, USA). Lanes consisting of 282 and 113 bp bands were considered to be the C/C genotype, lanes consisting of 282, 205 and 113 bp bands were considered the C/A genotype and lanes consisting of 282 and 205 bp bands were considered to be the A/A genotype.

### 2.4. Statistical Analysis

Statistical analysis was performed using SPSS software version 17.0 (Chicago, IL, USA). Student *t*-test was used to compare differences between continuous variables, and the χ^2^ test was used to compare differences between categorical variables. The Hardy–Weinberg equilibrium χ^2^ test was used to investigate genotype distribution deviation for both groups. Logistic regression was performed to compute the odds ratio (OR) and 95% confidence interval (95% CI) for prostate cancer development relative to the genotype. Multiple risk models were composed in order to inspect the mutual effect of different genes, alone or combined with acquired contributory factors, on risk of prostate cancer evolution. Age, hypertension and body mass index (BMI) were used as acquired contributory factors, as each of these factors was previously linked with an increased risk of cancer development [37,38,39]. The Kaplan–Meier test was utilized to calculate mean survival time and compute survival curves. A log-rank test was used to compare the mean survival time between carriers of different genotypes. *p* values < 0.05 were considered statistically significant.

## 3. Results

The demographic and clinical characteristics of the 475 study participants are shown in Table 1. No significant difference was observed with regard to age and BMI, whereas the presence of hypertension and diabetes mellitus type 2 was higher in the patient group than the control group (*p* < 0.05).

Genotype distributions, as well as the risk of prostate cancer development, are summarized in Table 2. As demonstrated, four risk models were computed: model 1 without any adjustments; model 2 with two other investigated genes as confounding factors; model 3 with age, hypertension and BMI as confounding factors; and model 4 with all combined. Carriers of at least one referent GPX1*C allele had no statistically significant increase in PC risk across all four examined models (OR 1 = 1.12, 95% CI = 0.59–2.13, *p* = 0.728; OR 2 = 1.06, 95% CI = 0.54–2.08, *p* = 0.869; OR3 = 1.25, 95% CI = 0.58–2.70, *p* = 0.570; OR4 = 1.27, 95% CI = 0.55–2.93, *p* = 0.570). Likewise, individuals with the Nrf2*C/C genotype had slightly increased PC risk, but it did not reach statistical significance. In contrast, individuals with at least one SOD2*C allele had an increased risk of PC development (OR = 1.22, 95% CI = 0.82–1.83, *p* = 0.326) compared to carriers of the SOD2*T/T genotype. This effect was significantly further amplified by the addition of confounding factors, from a 1.67-fold increase with acquired contributory factors in model 3 (OR = 1.67, 95% CI = 1.01–2.76, *p* = 0.046) to a 1.82–fold increase with both genetic and acquired factors in risk model 4 (OR = 1.82, 95% CI = 1.09–3.03, *p* = 0.022).

Furthermore, to assess whether genetic variants of *GPX1*, *SOD2* and *Nrf2* enzymes can mutually influence the risk for prostate development, the combined effect of gene polymorphisms was tested with and without previously stated confounding factors. Results are summarized in Table 3. Genetic variants that individually contributed to an increment in risk for disease occurrence were considered. The carriers of both SOD2*C/C or SOD2*C/T and Nrf2*C/C genotypes were at 2.5 times greater risk of the development of prostate cancer than individuals with SOD2*T/T and Nrf2*C/A or Nrf2*A/A genotypes, regardless of the presence of the GPX1*C allele (OR = 2.48, 95% CI = 0.82–1.83, *p* = 0.022; OR = 2.48, 95% CI = 0.87–1.98, *p* = 0.021; model 1 and model 2, respectively). This effect was enhanced by inclusion of other confounding factors, increasing risk from 3.75-fold in model 3 (OR = 3.75, 95% CI = 1.01–2.76, *p* = 0.009) to 4-fold in model 4 (OR = 4.07, 95% CI = 1.09–3.03, *p* = 0.006). The combined effect of the GPX1*C allele with either the SOD2*C allele or the Nrf2*C/C genotype showed no significant influence on PC development across all four investigated models (*p* > 0.05).

**Figure 1:**
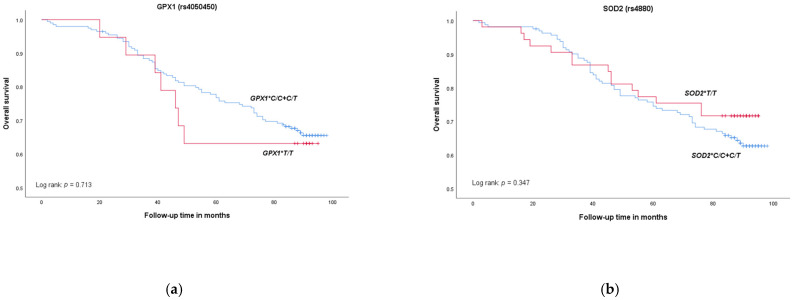

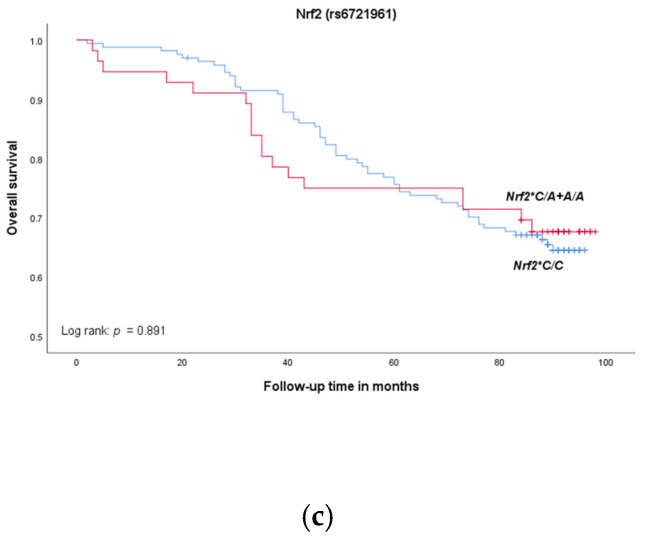
Overall survival in patients with prostate cancer according to (**a**) *GPX1* genetic variants, (**b**) *SOD2* genetic variants and (**c**) *Nrf2* genetic variants.

Thirteen patients were lost (5%) during the mean follow-up time of 75 ± 25 months (ranging from 2–98 months). Of the remaining 222 patients, 76 died (34%), and 146 (66%) were alive at the end of follow-up period. The overall survival of patients with prostate cancer with respect to *GPX1*, *SOD2* and *Nrf2* gene polymorphisms is presented in Figure 1. For *GPX1* and *SOD2* polymorphisms, survival time did not significantly differ in carriers of at least one referent allele GPX1*C or SOD2*C compared to variant homozygote carriers GPX1*T/T or SOD2*T/T (log rank = 0.135, *p* = 0.713; log rank = 0.886, *p* = 0.347; respectively). Similarly, there was no statistically significant difference in overall survival between carriers of at least one variant allele Nrf2*A compared to carriers of referent homozygote Nrf2*C/C (log rank = 0.019, *p* = 0.891).

## 4. Discussion

Several studies have demonstrated that small yet significant risk for the development and progression of prostate cancer has been associated with deleterious effects of certain polymorphisms found in genes encoding antioxidant enzymes, as well as their regulatory proteins [40,41,42,43]. In this case–control study, we found that a polymorphism found in the gene constituting immediate antioxidant defense, the SOD2*C allele (rs4880), was associated with increased risk of PC development. The risk was further amplified by the addition of both acquired and genetic confounding factors. In addition, when combined with the Nrf2*C/C genotype (rs6721961), the SOD2*C allele enhanced the risk of PC development from 2.48 times when investigated alone to 4.07 times when analyzed in combination with contributory factors.

*SOD2* gene polymorphisms have been extensively studied in malignant diseases, among which one of the most researched is rs4880. Previous reports demonstrated that individuals with the SOD2*C/C genotype are at increased higher risk of prostate cancer development [30,41,44,45], in accordance with the findings of our study. Moreover, a recent meta-analysis by Zhang et al. identified the SOD2*C allele as a significant contributor to PC risk. Furthermore, they identified an association between the downregulation of SOD2 expression with low levels of SOD2 and shorter disease-free survival [46]. In contrast, in another study that investigated patients with initially non-metastatic disease, *SOD2* rs4880 SNP was not reported to contribute to overall survival [47]. During our follow-up period, overall survival curves did not reach statistical significance in relation to the *SOD2* gene polymorphism. As previously mentioned, SOD plays a pivotal role in maintaining redox balance and protecting cells from ROS damage [48]. It is postulated that functional polymorphism *SOD2* rs4880 contributes to the difference in enzyme activity, with the SOD2*T variant associated with decreased mRNA expression and stability [49,50]. Interestingly, genetic studies that focused on the risk of malignant disease development reported increased risk in individual carriers of the SOD2*C allele, as mentioned above. One proposed explanation for these findings is that superoxide anion production can be mediated by proinflammatory ligands, such as interferon-gamma (IFN-γ), which activates the Janus kinase (JAK) signal transducer and activator of transcription (STAT) pathway and upregulates nicotinamide-adenine dinucleotide phosphate (NADPH) oxidase subunits and advanced glycation end products (AGEs) via the phosphoinositide-3-kinase (PI3)/mitogen-activated protein kinase (MAPK) pathway [51]. In a dismutase reaction, SOD2 produces H_2_O_2_, which functions as a secondary messenger. In overexpressed SOD2 cells, the increase in H_2_O_2_ can lead to malignant transformation, progression and even therapy resistance [52].

Previous research has established Nrf2 as a key regulatory antioxidant protein that binds to antioxidant response element (ARE) in the promotor region of target antioxidant genes [20,53]. To date, only one study has assessed the polymorphic expression of Nrf2 and identified a non-significant association of *Nrf2* rs10506328 polymorphism with prostate cancer [40]. The role of *Nrf2* rs6721961 SNP has not yet been studied in relation to susceptibility to prostate cancer development. This particular polymorphism is located in the gene promotor region controlling Nrf2 protein basal expression and self-induction [24,54,55]. In our study, we found no significant association between genetic variants and disease occurrence. However, in breast cancer patients (also a hormone-dependent cancer), the Nrf2*A/A genotype was associated with 4.6 times increased risk of disease development compared to Nrf2*C/C [56]. The same authors also showed that low-extent cytoplasmic Nrf2 protein expression correlated with the Nrf2*T allele among invasive breast cancer patients. This polymorphism has also been investigated in other urinary cancers, such as clear cell renal cell carcinoma (ccRCC), urinary bladder cancer and testicular cancer. In bladder cancer, *Nrf2* rs6721961 SNP exerted no influence with respect to risk of disease development [57]. Similar results were obtained in relation to testicular cancer. In particular, although the carriers of the Nrf2*C allele were at a near two-times increased risk of testicular cancer development, the risk was not statistically significant [58]. Recently, in a study conducted in Serbian patients, *Nrf2* SNP alone did not influence the risk of ccRCC development; however, the Nrf2*A allele in combination with the SOD2*T allele increased the risk of disease occurrence by almost threefold [13]. In contrast, in our study, the combination of the Nrf2*C/C genotype and the SOD2*C allele amplified the risk of PC development when compared to carriers of both the Nrf2*A allele and the SOD2*T/T genotype. Yamaguchi et al. observed that among ccRCC patients, the Nrf2*A allele was associated with increased protein expression, and in the metastatic setting, it corresponded with worse therapy response and unfavorable outcome [59].

GPX1 counters high H_2_O_2_ production by reducing it to H_2_O [60]. Functional SNP rs1050450 in the *GPX1* gene has been previously investigated in association with the risk of prostate cancer development. However, contradictory results have been reported. In particular, the meta-analysis by Men T. et al. demonstrated no significance in PC development among referent and variant allele carriers; they also reported an increase in bladder cancer occurrence in individuals with the GPX1*T allele [61]. In contrast, in a Macedonian population of PC patients, the GPX1*T allele played a protective role in disease development [62]. In this study, *GPX1* polymorphism did not contribute to PC risk or overall survival alone or in combination with other investigated gene polymorphisms across all models.

The present study is subject to some limitations that should be mentioned. Firstly, because only Caucasian males participated, these results probably cannot be transferred to a population that includes various ethnicities. Secondly, the follow-up period was short, given the long natural evolution of the disease, which could explain the absence of differences in terms of survival. Longer follow-up might help recognize statistically significant differences in overall survival in this study group. Thirdly, the study population was relatively small.

## 5. Conclusions

The *SOD2* (rs4880) gene polymorphism, alone or in combination with the *Nrf2* (rs6721961) gene polymorphism, could serve as a possible biomarker of prostate cancer development. Prostate cancer consists of multiple entities with varying tumor aggressiveness, clinical manifestation and prognosis. Identifying biomarkers that can contribute to detection of the disease at its early stage or recognizing those patients in need of earlier or more aggressive treatment is essential in enabling improved quality of life and longer survival in this population. We hope that this epidemiological study can serve as a basis for further in-depth research comprising various ethnicities with a longer follow-up period and a larger sample of participants.

## Figures and Tables

**Table 1:** Demographic and clinical characteristics of prostate cancer patients and controls.

Variable	Patients *n* = 235	Controls *n* = 240	*p*
Age (years) *	68.84 ± 6.95	67.43 ± 9.01	0.057
BMI (kg/m^2^) *	26.98 ± 3.48	26.71 ± 3.83	0.472
Hypertension, *n* (%) **			
Yes	127 (58)	82 (43)	
No	93 (42)	109 (57)	**0.003**
Diabetes mellitus type 2, *n* (%) **			
Yes	38 (17)	11 (7)	
No	190 (83)	154 (93)	**0.003**
Initial PSA (ng/mL), *n* (%)**			
<10	78 (34)	/	/
10–20	67 (29)	/	/
>20	85 (37)	/	/
Gleason score, *n* (%) **			
6	56 (30)	/	/
7 (3 + 4)	52 (28)	/	/
7 (4 + 3)	28 (15)	/	/
8	28 (15)	/	/
9/10	22 (12)	/	/
Treatment modality, *n* (%)			
Radical prostatectomy	97 (41)	/	/
EBRT	138 (59)	/	/

* mean ± SD; BMI—body mass index, PSA—prostate-specific antigen, EBRT—external beam radiation therapy; / not applicable; ** based on available information.

**Table 2:** *GPX1*, *SOD2* and *Nrf2* genotype distributions in PC patients and controls and the risk of disease development.

Genotype	Patients, *n* (%)	Controls, *n* (%)	OR 1 ** (95% CI)	*p*	OR 2 ** (95% CI)	*p*	OR 3 ** (95% CI)	*p*	OR 4 ** (95% CI)	*p*
*GPX1*										
GPX1*T/T	19 (8)	22 (9)	1.0		1.0		1.0		1.0	
GPX1*C/C + C/T	211 (92)	218 (91)	1.12 (0.59–2.13)	0.728	1.06 (0.54–2.08)	0.869	1.25 (0.58–2.70)	0.570	1.27 (0.55–2.93)	0.570
*SOD2*										
SOD2*T/T	60 (26)	73 (30)	1.0		1.0		1.0		1.0	
SOD2*C/C + C/T	168 (74)	167 (70)	1.22 (0.82–1.83)	0.326	1.31 (0.87–1.98)	0.191	**1.67 (1.01–2.76)**	**0.046**	**1.82 (1.09–3.03)**	**0.022**
*Nrf2*										
Nrf2*C/A + A/A	58 (25)	64 (28)	1.0		1.0		1.0		1.0	
Nrf2*C/C	176 (75)	166 (72)	1.18 (0.77–1.77)	0.457	1.17 (0.77–1.78)	0.468	1.24 (0.74–2.07)	0.417	1.28 (0.75–2.17)	0.362

** OR 1—crude results without confounding factors; OR 2—with two other genes as confounding factors; OR 3—with age, body mass index and hypertension as confounding factors; OR 4—with all previously stated confounding factors; 95% CI—95% confidence interval; *p* < 0.05 was considered statistically significant; data on successful genotypisation in both the case and control groups.

**Table 3:** Combined effects of gene polymorphisms on risk of PC development.

Genotype	Patients, *n* (%)	Controls, *n* (%)	OR 1 ** (95% CI)	*p*	OR 2 ** (95% CI)	*p*	OR 3 ** (95% CI)	*p*	OR 4 ** (95% CI)	*p*
*GPX1 + Nrf2*										
GPX1*T/T + Nrf2*C/A + A/A	3 (2)	3 (2)	1.0		1.0		1.0		1.0	
GPX1*C/C + C/T + Nrf2*C/C	156(98)	150 (98)	1.04 (0.21–5.23)	0.962	1.02 (0.20–5.15)	0.869	0.31 (0.03–3.79)	0.570	0.28 (0.02–3.83)	0.339
*SOD2 + Nrf2*										
SOD2*T/T + Nrf2*C/A + A/A	10 (8)	24 (17)	1.0		1.0		1.0		1.0	
SOD2*C/C + C/T + Nrf2*C/C	122 (92)	118(83)	**2.48 (0.82–1.83)**	**0.022**	**2.48 (0.87–1.98)**	**0.021**	**3.75 (1.01–2.76)**	**0.009**	**4.07 (1.09–3.03)**	**0.006**
*GPX1 + SOD2*										
GPX1*T/T + SOD2*T/T	4 (2)	7 (4)	1.0		1.0		1.0		1.0	
GPX1*C/C + C/T + SOD2*C/C + C/A	153 (98)	152 (96)	1.76 (0.50–6.14)	0.374	1.58 (0.44–5.72)	0.484	1.07 (0.19–6.17)	0.938	1.12 (0.19–6.50)	0.898

** OR 1—crude results without confounding factors; OR 2—with remaining genes presented in Table 3 as confounding factors; OR 3—with age, body mass index and hypertension as confounding factors; OR 4—with all previously stated confounding factors; 95% CI—95% confidence interval; *p* < 0.05 was considered statistically significant; data on successful genotypisation in both the case and control groups.

## Data Availability

The data supporting the reported results can be provided upon request in the form of datasets available at the Institute of Oncology and Radiology of Serbia, Clinic of Urology, University Clinical Centre of Serbia and the Institute of Medical and Clinical Biochemistry, Faculty of Medicine, University of Belgrade.

## Supplementary Materials

The following supporting information can be downloaded at: https://www.mdpi.com/article/10.3390/medicina58101414/s1, Figure S1: Histological image to support findings regarding Gleason grades 1 and 2; Figure S2: Histological image to support findings regarding Gleason grade 3; Figure S3: Histological image to support findings regarding Gleason grade 4; Figure S4: Histological image to support findings regarding Gleason grade 5.
