# Supplementary material for: The Association of Polymorphisms in Genes Encoding Antioxidant Enzymes GPX1 (rs1050450), SOD2 (rs4880) and Transcriptional Factor Nrf2 (rs6721961) with the Risk and Development of Prostate Cancer
Source: Medicina (Kaunas). 2022 Oct 9;58(10):1414. doi: 10.3390/medicina58101414 (PMC9611982; doi:10.3390/medicina58101414)
Supplement: Supplementary file 1 [file medicina-58-01414-s001.zip › medicina-1916936-supplementary.pdf]

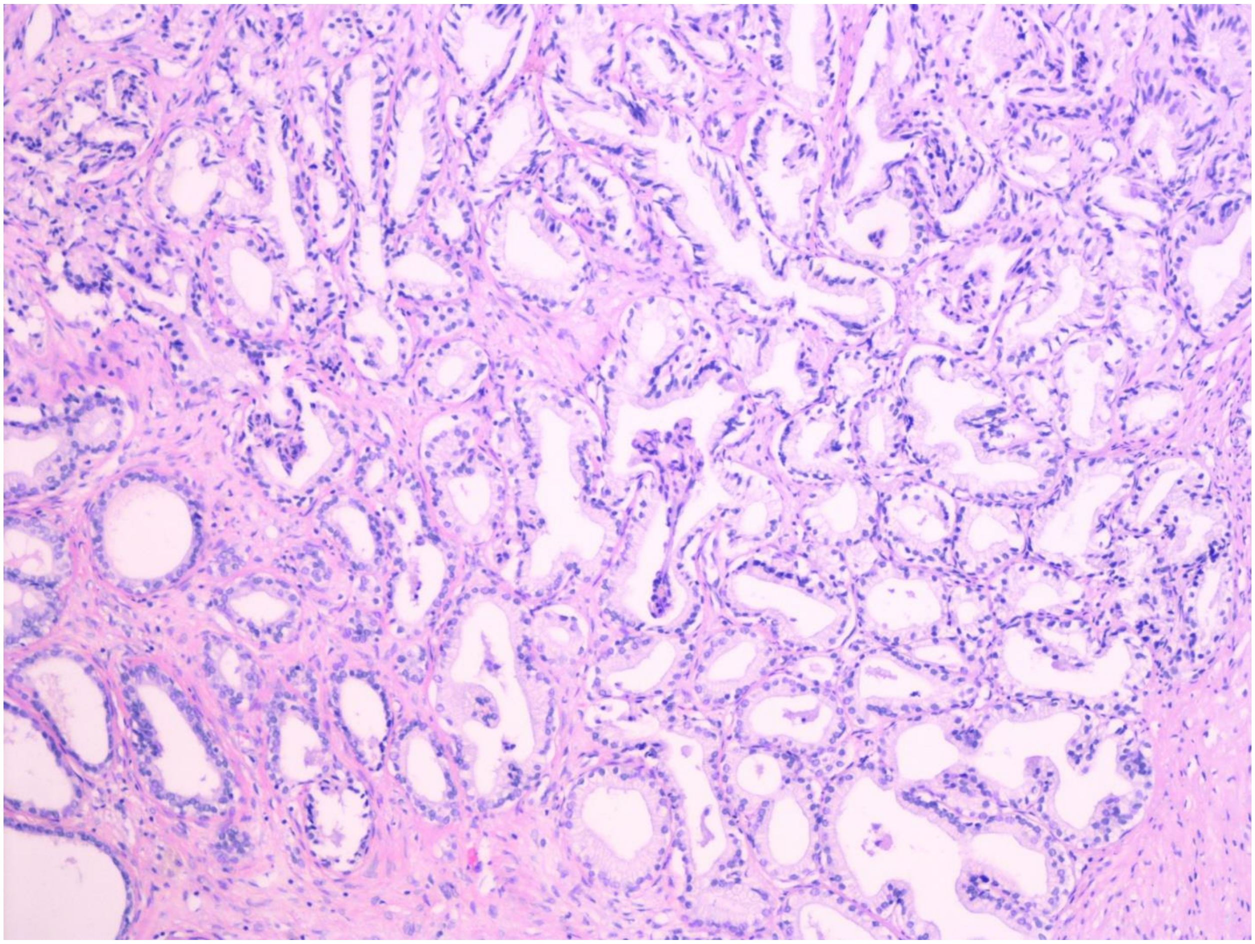

Figure S1. Histological image to support findings regarding Gleason 1 and 2

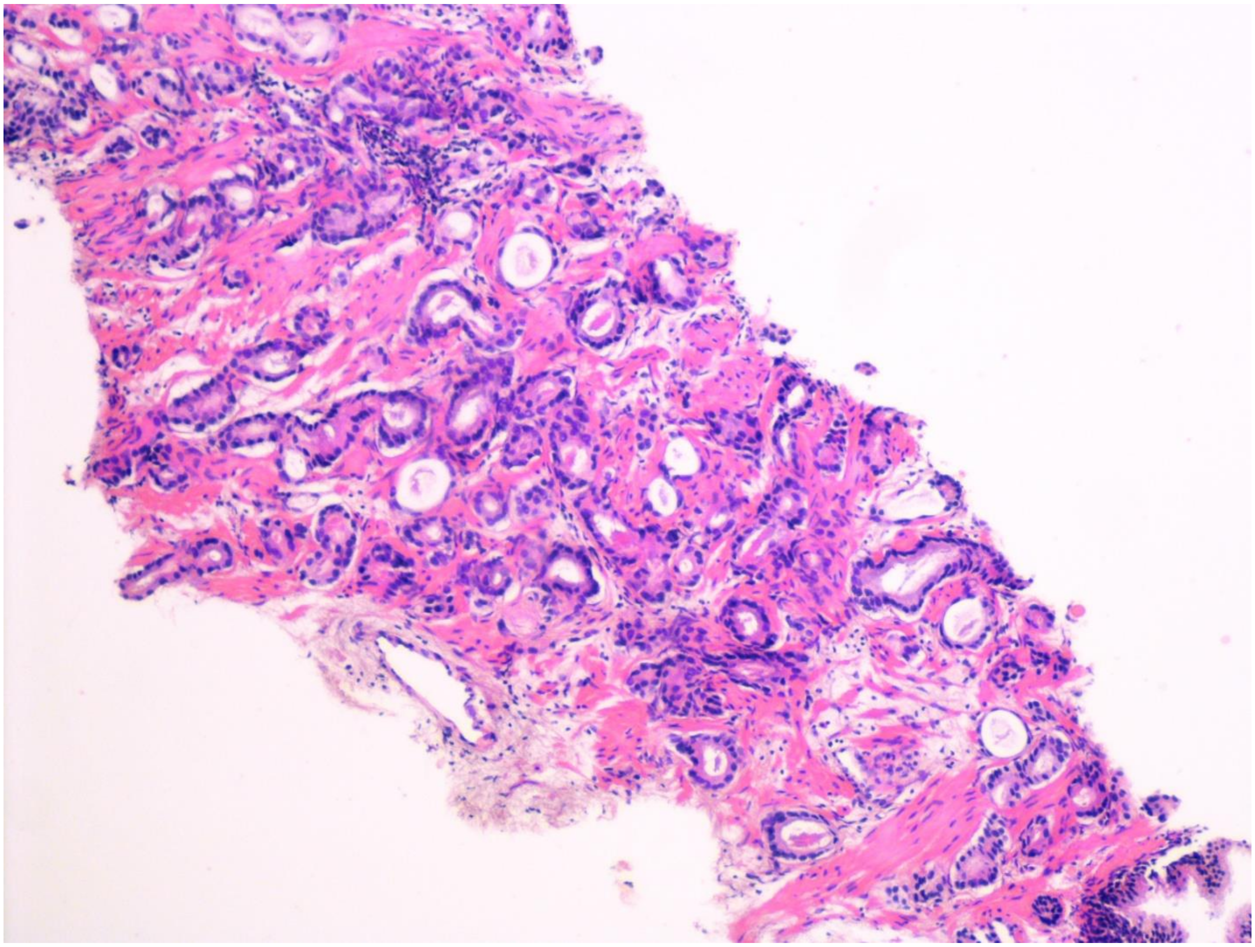

Figure S2. Histological image to support findings regarding Gleason 3

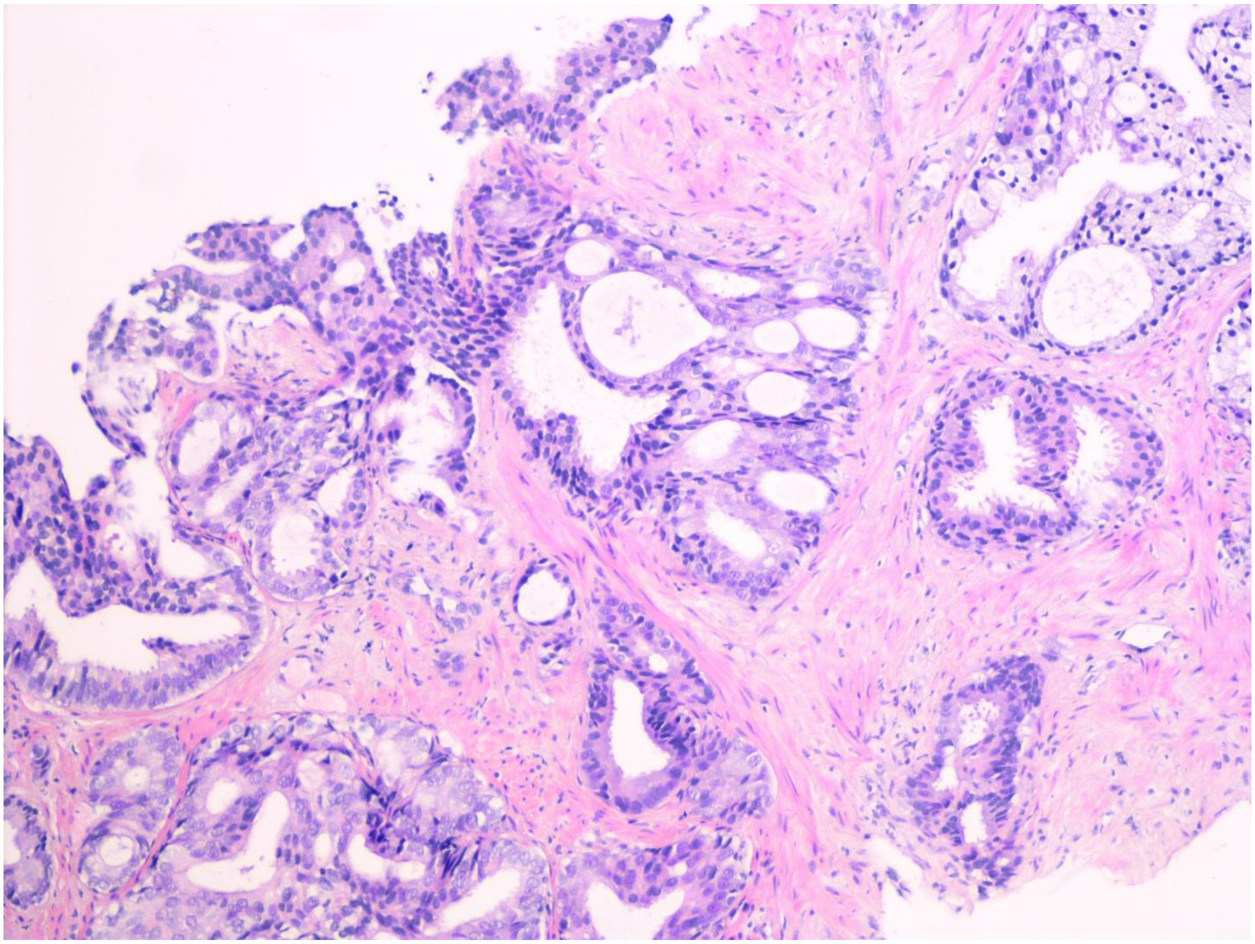

Figure S3. Histological image to support findings regarding Gleason 4

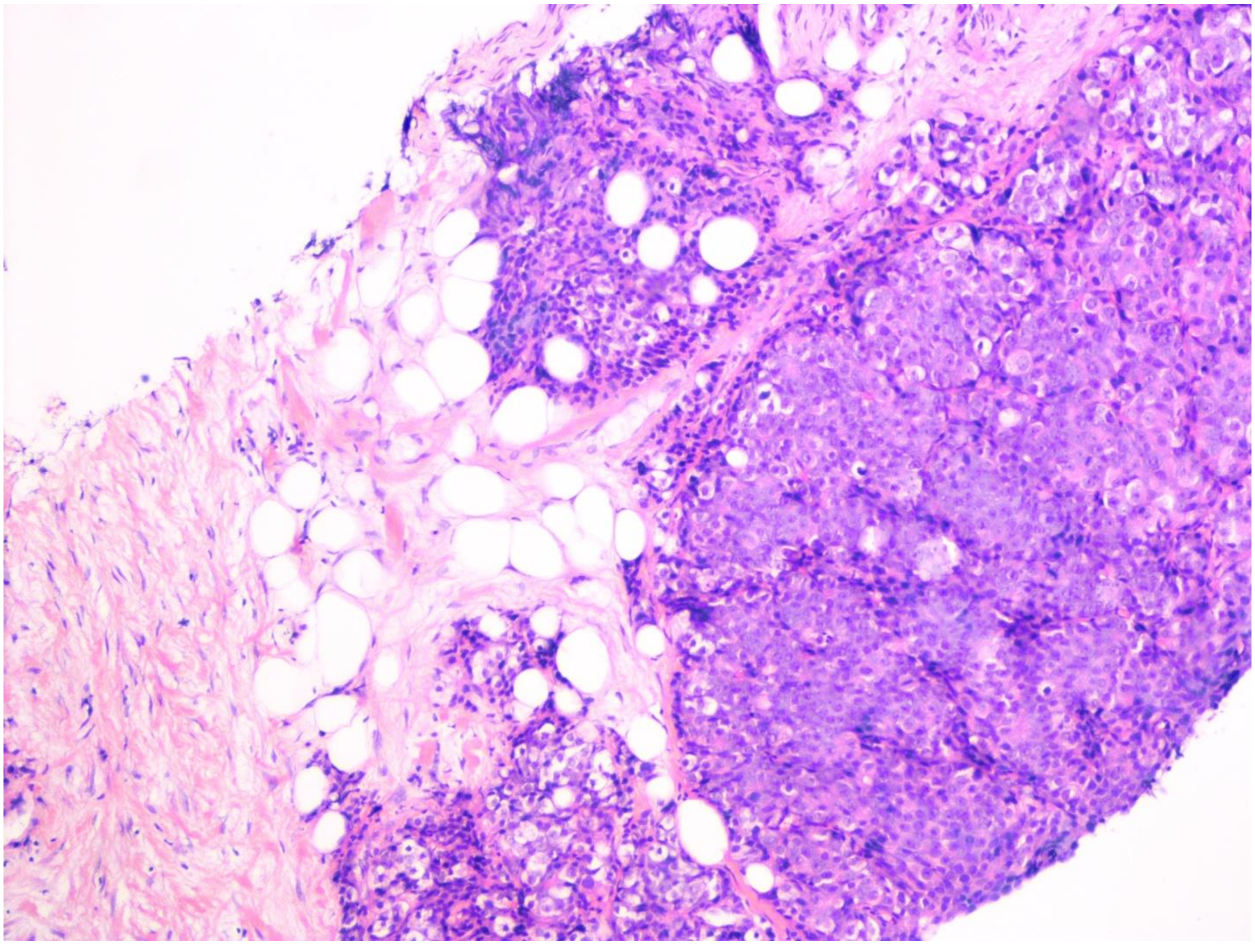

Figure S4. Histological image to support findings regarding Gleason 5
